# Supplementary material for: Adjuvant dendritic cell based immunotherapy (DCBI) after cytoreductive surgery (CRS) and hyperthermic intraperitoneal chemotherapy (HIPEC) for peritoneal mesothelioma, a phase II single centre open-label clinical trial: rationale and design of the MESOPEC trial
Source: BMJ Open. 2019 May 14;9(5):e026779. doi: 10.1136/bmjopen-2018-026779 (PMC6530331; doi:10.1136/bmjopen-2018-026779)
Supplement: Author's manuscript [file bmjopen-2018-026779.draft_revisions.pdf]

BMJ Open

BMJ Open is committed to open peer review. As part of this commitment we make the peer review history of every article we publish publicly available.

When an article is published we post the peer reviewers' comments and the authors' responses online. We also post the versions of the paper that were used during peer review. These are the versions that the peer review comments apply to.

The versions of the paper that follow are the versions that were submitted during the peer review process. They are not the versions of record or the final published versions. They should not be cited or distributed as the published version of this manuscript.

BMJ Open is an open access journal and the full, final, typeset and author-corrected version of record of the manuscript is available on our site with no access controls, subscription charges or pay-per-view fees (http://bmjopen.bmj.com).

If you have any questions on BMJ Open's open peer review process please email info.bmjopen@bmj.com

BMJ Open

Adjuvant dendritic cell based immunotherapy (DCBI) after cytoreductive surgery (CRS) and hyperthermic intraperitoneal chemotherapy (HIPEC) for peritoneal mesothelioma, a single center open label clinical trial. Rationale and design of the MESOPEC trial.

Journal:	*BMJ Open*
Manuscript ID	bmjopen-2018-026779
Article Type:	Protocol
Date Submitted by the Author:	19-Sep-2018
Complete List of Authors:	de Boer, Nadine; Erasmus MC, Surgical Oncology<br>van Kooten, Job; Erasmus MC, Surgical Oncology<br>Burger, Jacobus; Erasmus MC, Surgical Oncology<br>Aerts, Joachim; Erasmus MC, Pulmonary Medicine<br>Verhoef, Cornelis; Erasmus MC, Surgical Oncology<br>Madsen, Eva; Erasmus MC, Surgical Oncology
Keywords:	Malignant peritoneal mesothelioma, Cytoreductive surgery and hyperthermic intraperitoneal chemotherapy, Dendritic cell based immunotherapy

SCHOLARONE™
Manuscripts

MESOPEC-Trial

Adjuvant dendritic cell based immunotherapy (DCBI) after cytoreductive surgery (CRS) and hyperthermic intraperitoneal chemotherapy (HIPEC) for peritoneal mesothelioma, a single center open label clinical trial. Rationale and design of the MESOPEC trial.

N.L. de Boer[a], J.P. van Kooten[a], J.W.A. Burger[a], J.G.J.V. Aerts[b], C. Verhoef[a], E.V.E. Madsen[a]

a. Department of Surgical Oncology, Erasmus University Medical Center, Rotterdam, the Netherlands
b. Department of Pulmonary Medicine, Erasmus University Medical Center, Rotterdam, the Netherlands

Corresponding author:
N.L. de Boer
Erasmus MC
Department of surgical oncology
Dr. Molenwaterplein 40
3015 GD, Rotterdam, the Netherlands
n.deboer@erasmusmc.nl
(+31)010-704 21 25

Word count: 2831

Keywords: Malignant peritoneal mesothelioma, Cytoreductive surgery and hyperthermic intraperitoneal chemotherapy, Dendritic cell based immunotherapy

For peer review only

SPIRIT guidelines data set for clinical trials

Primary registry and trial identifying number	EudraCT number: 2017-000897-12<br>Netherlands Trial Register: NTR7060
Date of registration in primary registry	October 2017
Source(s) of monetary or material support	Erasmus University Medical Center, Rotterdam, the Netherlands<br>Dutch Cancer Society (KWF Kankerbestrijding)<br>Stichting Coolsingel
Primary sponsor	Erasmus University Medical Center, Rotterdam, the Netherlands
Secondary sponsors	Dutch cancer society (KWF Kankerbestrijding)<br>Stichting Coolsingel
Contact for public queries	N.L. de Boer, study coordinator<br>Department of surgical oncology<br>Erasmus MC, Rotterdam, the Netherlands<br>n.deboer@erasmusmc.nl, (+31)010-704 21 25<br><br>J.P. van Kooten, study coordinator<br>Department of surgical oncology<br>Erasmus MC, Rotterdam, the Netherlands<br>j.kooten@erasmusmc.nl, (+31)010-704 21 25
Contact for scientific queries	E.V.E. Madsen, principal investigator<br>Department of surgical oncology<br>Erasmus MC, Rotterdam, the Netherlands<br>e.madsen@erasmusmc.nl, (+31)010-704 10 82
Public title	Adjuvant dendritic cell based immunotherapy (DCBI) after cytoreductive surgery (CRS) and hyperthermic intraperitoneal chemotherapy (HIPEC) for peritoneal mesothelioma: the MESOPEC-trial.
Scientific title	Adjuvant dendritic cell based immunotherapy (DCBI) after cytoreductive surgery (CRS) and hyperthermic intraperitoneal chemotherapy (HIPEC) for peritoneal mesothelioma. An open-label single center phase II clinical trial.
Countries of recruitment	The Netherlands
Health conditions or problems studied	Malignant peritoneal mesothelioma
Interventions	Vaccination with autologous dendritic cells loaded with allogeneic mesothelioma specific tumor antigens, after standard care (CRS-HIPEC)
Key inclusion and exclusion criteria	Inclusion Criteria:<br>Confirmed diagnosis of epithelial peritoneal mesothelioma.

For peer review only

	WHO-ECOG performance status 0-1, expected survival at least six months.<br>Adequate organ function and bone marrow reserves.<br>Positive delayed type hypersensitivity skin test for positive control antigen.<br><br>Exclusion criteria:<br>Extra-abdominal disease/metastatic disease.<br>Current use of steroids or other immunosuppressive agents.<br>Prior cytoreductive surgery.<br>Prior malignancy other than basal cell carcinoma within ten years of inclusion.<br>Patients with a known allergy to shellfish<br>Serious chronic or acute illness considered to constitute unwarranted high risk for CRS-HIPEC or dendritic cell treatment.<br>Pregnant or lactating women.
Study type	Open label single center phase II study
Date of first enrolment	March 2018
Target sample size	20
Recruitment status	Recruiting
Primary outcome	Feasibility (DCBI therapy is considered feasible when 75% of patients enrolled in this study are able to receive and finish dendritic cell vaccination after CRS-HIPEC)
Key secondary outcome(s)	Safety<br>Immunologic response after dendritic cell vaccination

For peer review only

ABSTRACT

Introduction: Malignant peritoneal mesothelioma (MPM) is an uncommon but aggressive neoplasm and has a strong association with asbestos exposure. MPM has low survival rates of approximately one year even after (palliative) surgery and/or systemic chemotherapy. Recent advances in treatment strategies focusing on cytoreductive surgery (CRS) and hyperthermic intraperitoneal chemotherapy (HIPEC) have resulted in improved median survival of 53 months and a 5-year survival of 47%. However, recurrence rates are high. Current systemic chemotherapy in the adjuvant setting is of limited efficacy, while immunotherapy with dendritic cell based immunotherapy (DCBI) has yielded promising results in murine models with peritoneal mesothelioma and in patients with pleural mesothelioma.

Methods and analysis: The MESOPEC trial is an open-label single center phase II study. The study population are adult patients with histological/cytological confirmed diagnosis of epithelioid malignant peritoneal mesothelioma. Intervention: four to six weeks before CRS-HIPEC a leukapheresis is performed of which the monocytes are used for differentiation to dendritic cells (DCs). Autologous DCs pulsed with allogeneic tumor associated antigens (MesoPher) are re-injected eight to ten weeks after surgery, three times biweekly. Additional booster vaccinations are given at three and six months.

Primary objective is to determine the feasibility of administering DCBI after CRS-HIPEC in patients with malignant peritoneal mesothelioma. Secondary objectives are to assess safety of DCBI in patients with peritoneal mesothelioma and determine whether a specific immunologic response against the tumor occurs as a result of this adjuvant immunotherapy.

Ethics and dissemination: Permission to carry out this study protocol has been granted by the Central Committee on Research Involving Human Subjects (CCMO in Dutch) and the Research Ethics Committee (METC in Dutch). The results of this trial will be submitted for publication in a peer-reviewed journal.

Registration: Netherlands Trial Register: NTR7060. EudraCT: 2017-000897-12.

For peer review only

Strengths and limitations of this study

- The dendritic cell vaccines used in this protocol can be manufactured on a large scale, because autologous dendritic cells are loaded with allogeneic tumor associated antigens.
- Dendritic cell based immunotherapy has shown to have very limited side effects, especially when compared to systemic chemotherapy.
- This study will provide clinicians and scientists with important information about the immunologic response after dendritic cell vaccination
- Since all patients undergo CRS-HIPEC prior to DCBI therapy, the effect of DCBI treatment must be determined by assessing the immune response and overall clinical condition of each patient.
- In this phase II clinical trial the effect of DCBI on disease free and overall survival cannot be determined, when DCBI is considered safe and feasible, a phase III clinical trial will be conducted to determine the effect on survival.

For peer review only

1. INTRODUCTION

Malignant peritoneal mesothelioma (MPM) is a highly lethal neoplasm, arising from the serosal lining of the peritoneal cavity. It has a strong association with exposure to asbestos. Non-specific clinical symptoms like weight loss, abdominal pain and distension, contribute to a delay in diagnosis. As a result, the majority of MPM cases are identified at an advanced stage, creating an overall poor life-expectancy of 4-12 months if left untreated.(1) Even after (palliative) surgery and/or systemic chemotherapy, MPM has poor survival of approximately one year.

In recent years treatment focus has shifted towards a more aggressive approach, utilizing cytoreductive surgery (CRS) and hyperthermic intraperitoneal chemotherapy (HIPEC). Patients that underwent this treatment, had a better prognosis with median survival of 53 months and 5-year survival of 47%. (2) However, even after CRS-HIPEC recurrence rates are high with reported median progression-free survival and disease-free survival ranging from 11 to 28 months and 7.2 to 40 months respectively.(1) One explanation is that it is difficult to perform complete cytoreduction, as MPM often grows diffusely throughout the abdominal cavity.(3) But even when macroscopic complete cytoreduction is reached, loco-regional recurrence is often seen. A study that included 108 patients in whom complete or near-complete cytoreduction was achieved, showed local recurrence in 38% of patients after median follow up of 48.8 months.(4)

Effective adjuvant therapies are pressingly needed for abovementioned reasons. Dendritic cell based immunotherapy (DCBI) has shown promising results by harnessing the potency and specificity of the immune system. The first DCBI for mesothelioma was developed in the Erasmus MC Rotterdam and has been tested in murine models with peritoneal mesothelioma and in clinical phase I/II studies for patients with pleural mesothelioma.(5-11) These studies have shown that DCBI induces durable responses and higher survival rates compared to the general mesothelioma population. DC-therapy was well tolerated in these patients without grade 3 or 4 toxicities. Only low grade fever and flu-like symptoms (grade 1-2), were seen for 24 hours after treatment. In a dose escalation phase I trial, the safety of using allogeneic tumor lysate (PheraLys) for the loading of the DCs was assessed. PheraLys is a tumor cell lysate derived from 5 well-characterized cell lines from patients with malignant mesothelioma. Tumor lysate priming strategies may be advantageous in providing the full antigenic repertoire of the tumor and might reduce the possibility of tumor escape by inducing a broader immune response. In this study adverse events were similar to earlier studies and importantly, no severe adverse events were observed. Furthermore, clinical responses were established radiographically and some long time survivors are being observed.

Previous preclinical studies demonstrated that DCBI has the capacity to slow down tumor growth, although tumor load has an important role in survival.(12) Mice had a better outcome when DCs were injected early in tumor development.(5) Mesothelioma cells produce specific cytokines and attract regulatory T-cells that

For peer review only

suppress efficient immune responses, indicating that patients with low tumor load have a better functioning immune system and better anti-tumor responses.(13) Therefore in this trial DCBI is given as an adjuvant treatment after complete macroscopic cytoreduction and HIPEC.

Main objective of this clinical trial is to determine feasibility of administering adjuvant DCBI after CRS-HIPEC. Secondary objectives are to asses safety and determine if a specific immunologic response against the tumor occurs after DC therapy. When DCBI is considered safe and feasible as adjuvant treatment for patients with MPM, further research (phase III study) is warranted to determine the effect on survival.

2. METHODS AND ANALYSIS

2.1 Study Design

2.1.1 Study design

The MESOPEC trial is an open-label, single arm, single center phase II clinical trial. All patients included in this trial will receive adjuvant DCBI after CRS-HIPEC.

2.1.2 Patient timeline and additional procedures

Four to six weeks before surgery patients will undergo leukapheresis for dendritic cell vaccine production purposes. At baseline, subjects undergo CRS-HIPEC. At six weeks after surgery, the investigators will determine if the patient is sufficiently recovered and fit to undergo DCBI. Dendritic cell vaccinations will be given at eight to ten weeks after surgery three times biweekly. After the third vaccination, delayed type hypersensitivity (DTH) skin test is performed. When DTH skin test result is positive for the dendritic cell vaccine, a 3 mm skin biopsy will be taken for further analyses. At three and six months after the first vaccination, two additional booster vaccinations will be given. Additional to study-related treatment, patients receive standard care and follow up required after CRS-HIPEC.

Total duration of the treatment protocol is eight to nine months. In total, 11 additional visits are required to adhere to the study protocol. For the production of DCBI patients have to undergo leukapheresis. For the purpose of immune-monitoring, additional blood samples will be collected at seven moments during the study. (fig.1)

2.1.3 Study population

The study population consists of adult patients diagnosed with MPM. In order to participate in the study, patients must meet the following inclusion criteria:

- Histologically or cytological confirmed diagnosis of epithelioid MPM
- WHO-ECOG performance status of 0 or 1

For peer review only

- Normal organ function and adequate bone marrow reserve (absolute neutrophil count >1.0 $*10^9$/l, platelet count >100 $*10^9$/l, Hb >6.0 mmol/l)
- Positive DTH skin test against at least one positive control antigen tetanus toxoid
- Planned start date of vaccination within eight to ten weeks after CRS-HIPEC
- Expected survival prior to surgery must be at least six months
- At least 18 years of age, written informed consent according to ICH-GCP, ability to return to the study center for adequate follow up

A potential participant who meets any of the following criteria will be excluded from participation in the study:

- Extra abdominal mesothelioma (i.e. metastatic disease)
- Prior cytoreductive surgery
- Prior malignancy other than basal cell carcinoma within ten years of inclusion
- Serious concomitant disease or infection, including HIV or chronic viral hepatitis
- Current use of steroids or other immunosuppressive agents (at least six weeks discontinuation before the first vaccine, with exception of prophylactic usage of dexamethasone during chemotherapy).
- History of auto-immune disease or organ allografts
- Any disease that is considered to constitute an unwarranted high risk for CRS-HIPEC by the surgeon or study coordinator
- Pregnant or lactating women

2.1.4 Dendritic cell vaccine production

Dendritic cells are derived from peripheral blood mononuclear cells (PBMCs), by differentiating monocytes towards immature dendritic cells using specific cytokines. Immature dendritic cells are known for their high antigen uptake potential. Therefore they are exposed to tumor specific antigens (TAA) in a co-culture with allogeneic mesothelioma cell lysate. This lysate (PheraLys) is derived from five well specified mesothelioma cell lines. After exposure to PheraLys the immature dendritic cells are differentiated towards mature dendritic cells, which are ready to activate the immune system in vivo (MesoPher).

2.2 Objectives and Analysis

Primary objective

Primary objective is to determine feasibility of using DCBI after CRS-HIPEC in patients with peritoneal mesothelioma. DCBI after CRS-HIPEC is deemed feasible when at least 75% of patients that are included in this trial complete the full treatment schedule. This cut-off is based on the fact that currently around 75% of patients undergoing CRS-HIPEC are fit to undergo adjuvant therapy, such as systemic chemotherapy.

Secondary objectives

For peer review only

Secondary objectives are to asses safety of DCBI therapy after CRS-HIPEC and determine whether a specific immunologic response occurs due to dendritic cell vaccination

Safety

Previous clinical studies have shown that injection with tumor lysate-pulsed autologous DCs was overall well tolerated without systemic toxicity, with the exception of low-grade flu-like symptoms like fever and rigors. No grade 3 or 4 toxicity was observed.(11) However, safety and tolerability after major surgery has yet to be determined.

The administration of autologous cells that have been loaded with allogeneic human materials is a potential health risk. Because not the lysate itself is administered to the patients, but only after it has been processed by autologous dendritic cells, risks are expected to be limited.

The necessary sample size for the detection of grade 3 toxicity is calculated to be 20 patients (fig.3). All adverse events (AEs), serious adverse events (SAEs) and suspected unexpected serious adverse reactions (SUSARS) will be monitored and reported by the sponsor.

Immune response

Assessment of immune responses will be conducted on three levels in all treated patients; 1. responses that mark successful vaccination, 2. enhanced frequencies of tumor-specific T cells in peripheral blood samples, and 3. frequency shifts in other immune cell subsets.

1. Keyhole Limpet Hemocyanin (KLH) is added to the vaccines as a surrogate marker. With the use of KLH, we will assess whether an immune response against the vaccine has occurred and whether this response persists. KLH is known to induce a specific adaptive immune response readily detectable in sera (antibody response) and skin tests (cellular response) of vaccinated individuals. Serum samples will be collected before, during and after DCBI as well as at selected intervals during follow-up. Humoral responses to KLH will be detected using enzyme-linked immune sorbent assay (ELISA). Furthermore, patients will undergo a delayed type hypersensitivity (DTH) skin test before and after DCBI. DTH responses will be evaluated for local inflammation after 48 hrs and 3 mm punch biopsies will be collected in case inflammation occurs. These biopsies will subsequently be used for in situ immunostainings of i.e. DC, myeloid derived suppressor cells (MDSC), and CD8+ T cells.

2. To assess vaccine-induced frequencies of tumor-specific T cells, we will conduct a potency assay in accordance with the “Guideline on potency testing of cell based immunotherapy medicinal products for the treatment of cancer” as provided by the European Medicine Agency (EMA) in 2016 (EMA/CHMP/BWP/271475/2006 rev.1). The proposed assay can shortly be described as a co-culture of T cells isolated from pre- and post-treatment peripheral blood samples with autologous DCs loaded with autologous tumor lysate. Subsequently, we will measure T cell proliferation and activation markers via multicolor flow cytometry.

For peer review only - http://bmjopen.bmj.com/site/about/guidelines.xhtml

3. Phenotypical analysis of immune cell subsets will be conducted using flow cytometry to detect vaccine-induced changes in the frequencies of >100 immune cell(subsets) that represent distinct lineages and/or express different levels of activation, differentiation and co-signaling markers . Staining of fresh patient material at different time points will allow enumeration of different immune cells throughout therapy. Subsequent bulk analysis of frozen material focusses solely on an extensive array of T cell, MDSC and DC markers. Combination of these readouts allows for the generation of immune profiles for individual patients. The analysis of these profiles in turn will allow for the determination of prospective markers and better stratification of patient populations suited for vaccination therapy.

Statistical analysis

The statistical analyses/data summaries will be performed using SPSS. Other tools may be used for exploratory summaries and graphical presentations.

Primary endpoint is to determine the feasibility of administering DCBI after CRS-HIPEC in patients with malignant peritoneal mesothelioma. Based on previous results it is known that from all patients with colorectal carcinoma that underwent CRS-HIPEC about 25% is not able to receive adjuvant systemic therapy due to postoperative complications. Feasibility is set if 15 of 20 patients (75%) were able to undergo leukapheresis successfully, production of PheraLys and dendritic cell vaccines was successful and when patients were able to complete the full adjuvant treatment schedule.

3. ETHICS AND DISSEMINATION

Permission to implement this study protocol has been granted by the Central Committee on Research Involving Human Subjects (CCMO in Dutch) and the Research Ethics Committee (METC in Dutch) of the Erasmus MC. The study will be conducted in compliance with the ‘Medical Research Involving Human Subjects Act’ (WMO) and according to the principles of the Declaration of Helsinki (64th World Medical Association General Assembly, Fortaleza, Brazil, October 2013).

To generate more awareness of this current study and to increase referrals of potential study candidates to the Erasmus MC, a short Dutch summary of the study will be submitted to The Dutch Journal for Oncology (NTVO in Dutch). Also, presentations about the study have been given at the Dutch Society of Surgery meeting and the Peritoneal Surface Oncology Group International meeting in Paris.

The results of this clinical trial will be submitted for publication in a peer-reviewed scientific journal.

The investigator will permit auditors to carry out site visits to audit the compliance with regulatory guidelines. Similar auditing procedures may be conducted by agents of any regulatory body reviewing the results of this study.

For peer review only

The sponsor will submit yearly safety report to the accredited METC, competent authority, and competent authorities of the concerned Member States. This safety report consists of: (1) A list of all suspected (unexpected or expected) serious adverse reactions. (2) a report concerning the safety of the subjects, consisting of a complete safety analysis and an evaluation of the balance between the efficacy and the harmfulness of the medicine under investigation.

Within one year after the end of the study, the investigator/sponsor will submit a final study report with the results of the study, including any publications/abstracts of the study, to the accredited METC and the Competent Authority.

Currently three patients are included in the study protocol. Final patient is expected to be included at the end of 2020. First results are expected in 2021.

4. DISCUSSION

The main objective of the MESOPEC trial is to determine feasibility of DCBI as adjuvant treatment after CRS-HIPEC in patients with MPM. Secondary objectives are to assess safety and to monitor the immune response after DCBI. The MESOPEC trial is the first clinical trials offering adjuvant dendritic cell immunotherapy to patients with MPM. So far (neo)adjuvant systemic chemotherapy has shown no benefit on surgical or oncological outcome.(14) Systemic chemotherapy, using cisplatin and pemetrexed, is standard treatment in pleural mesothelioma and has been applied to patients with peritoneal mesothelioma. This has shown limited efficacy, considerable toxicity and even mortality, making it unfit for the treatment of MPM. (15)

The DCBI used in this current trial consists of personalized dendritic cell vaccines, produced with autologous dendritic cells that are loaded with tumor associated antigens derived from allogeneic tumor cell lysate. This treatment approach has multiple advantages. The biggest advantage of this strategy is that it is possible to produce personalized anti-cancer vaccines on a scale sufficient for clinical implementation in larger groups of patients. Another advantage is that so far DCBI has shown no severe side effects and therefore has little morbidity especially when compared to current other adjuvant treatment options, like systemic chemotherapy.

Analyses of tissue and blood samples that are collected throughout the study will provide valuable information for scientists and clinicians regarding the immunologic response after DCBI. Furthermore, potential of DCBI can be tested in vitro by culturing tumor cell lines derived from tumor tissue obtained during CRS. By doing so, in the future patients that will respond to immunotherapy can be identified before starting treatment.

Currently, CRS-HIPEC is the golden standard for a selected group of patients suffering from MPM. Eligibility for CRS-HIPEC is dependent on several clinical aspects such as 'peritoneal carcinoma index' (PCI), histological subtype and overall patient health condition. Previous studies have shown that outcome after CRS-HIPEC strongly depends on the completeness of cytoreduction.(1) Unfortunately it is very difficult to achieve

For peer review only - http://bmjopen.bmj.com/site/about/guidelines.xhtml

complete cytoreduction in patients with high PCI-score. It has been reported by others that immunotherapy is possibly more effective in patients with low tumor load. Therefore, in this study DCBI is given as adjuvant treatment after CRS-HIPEC. However, if a significant clinical effect can be achieved, in the future DCBI might be used as a neoadjuvant therapy making CRS-HIPEC with complete cytoreduction available for a larger number of patients.

We acknowledge the fact that this study has limitations. One limitation of this study is the small number of patients that will be included. Given the rarity of MPM, it is difficult to include large numbers of patients. However the sample size of this current study, should be sufficient to determine the feasibility and safety of adjuvant DCBI after CRS-HIPEC.

Another limitation of this study design is that it is not possible to determine radiological response to DCBI treatment. After debulking surgery, MPM will not be detectable on CAT-scans. Therefore the response to DCBI treatment must be determined by assessing the immune response and overall clinical condition of each patient. Clinical effect of DCBI on overall survival can only be determined after a longer period of follow up.

If DCBI is considered feasible as adjuvant treatment for MPM, a larger phase III clinical trial should be conducted to determine the effect on surgical and oncological outcome. Because MPM incidence in the Netherlands alone is very low, this clinical trial would have to be conducted internationally.

5. AUTHORS CONTRIBUTIONS

NB and JK drafted this manuscript. NB, JB, JA, CV and EM drafted the original study protocol and revised the manuscript. CV, JA and EM initiated the trial and supervised the drafting of the study protocol and manuscript. EM acquired funding for implementation of the trial protocol and is the primary clinical investigator. All authors approved the final manuscript.

6. COMPETING INTERESTS

None declared.

7. FUNDING STATEMENT

This work was supported by: The Dutch Cancer Society (in Dutch: KWF Kankerbestrijding), grant number: 10246,

Stichting Coolsingel, grant number: 482

8. REFERENCES

1. Helm JH, Miura JT, Glenn JA, et al. Cytoreductive surgery and hyperthermic intraperitoneal chemotherapy for malignant peritoneal mesothelioma: a systematic review and meta-analysis. Ann Surg Oncol. 2015;22(5):1686-93.

For peer review only

2. Yan TD, Deraco M, Baratti D, et al. Cytoreductive surgery and hyperthermic intraperitoneal chemotherapy for malignant peritoneal mesothelioma: multi-institutional experience. J Clin Oncol. 2009;27(36):6237-42.
3. Yan TD, Welch L, Black D, et al. A systematic review on the efficacy of cytoreductive surgery combined with perioperative intraperitoneal chemotherapy for diffuse malignancy peritoneal mesothelioma. Ann Oncol. 2007;18(5):827-34.
4. Baratti D, Kusamura S, Cabras AD, et al. Diffuse malignant peritoneal mesothelioma: long-term survival with complete cytoreductive surgery followed by hyperthermic intraperitoneal chemotherapy (HIPEC). Eur J Cancer. 2013;49(15):3140-8.
5. Hegmans JP, Hemmes A, Aerts JG, et al. Immunotherapy of murine malignant mesothelioma using tumor lysate-pulsed dendritic cells. Am J Respir Crit Care Med. 2005;171(10):1168-77.
6. Hegmans JP, Veltman JD, Lambers ME, et al. Consolidative dendritic cell-based immunotherapy elicits cytotoxicity against malignant mesothelioma. Am J Respir Crit Care Med. 2010;181(12):1383-90.
7. Veltman JD, Lambers ME, van Nimwegen M, et al. Low-dose cyclophosphamide synergizes with dendritic cell-based immunotherapy in antitumor activity. J Biomed Biotechnol. 2010;2010:798467.
8. Cornelissen R, Heuvers ME, Maat AP, et al. New roads open up for implementing immunotherapy in mesothelioma. Clin Dev Immunol. 2012;2012:927240.
9. Heuvers ME, Wisnivesky J, Stricker BH, et al. Generalizability of results from the National Lung Screening Trial. Eur J Epidemiol. 2012;27(9):669-72.
10. Cornelissen R, Lievense LA, Heuvers ME, et al. Dendritic cell-based immunotherapy in mesothelioma. Immunotherapy. 2012;4(10):1011-22.
11. Cornelissen R, Hegmans JP, Maat AP, et al. Extended Tumor Control after Dendritic Cell Vaccination with Low-Dose Cyclophosphamide as Adjuvant Treatment in Patients with Malignant Pleural Mesothelioma. Am J Respir Crit Care Med. 2016;193(9):1023-31.
12. Anguille S, Smits EL, Lion E, et al. Clinical use of dendritic cells for cancer therapy. Lancet Oncol. 2014;15(7):e257-67.
13. Hegmans JP, Hemmes A, Hammad H,et al. Mesothelioma environment comprises cytokines and T-regulatory cells that suppress immune responses. Eur Respir J. 2006;27(6):1086-95.
14. Deraco M, Baratti D, Hutanu I, et al. The role of perioperative systemic chemotherapy in diffuse malignant peritoneal mesothelioma patients treated with cytoreductive surgery and hyperthermic intraperitoneal chemotherapy. Ann Surg Oncol. 2013;20(4):1093-100.
15. Janne PA, Wozniak AJ, Belani CP, et al. Open-label study of pemetrexed alone or in combination with cisplatin for the treatment of patients with peritoneal mesothelioma: outcomes of an expanded access program. Clin Lung Cancer. 2005;7(1):40-6.

For peer review only

Fig.1 *Patient timeline. In total 11 additional visits are required. After informed consent is acquired, screening will take place in the form of full examination and DTH skintest. When patients comply to all criteria, they will undergo leukapheresis for production of dendritic cell vaccine. After two to four weeks patients undergo CRS-HIPEC. Eight to ten weeks after surgery, first vaccination is given, followed by two more vaccinations biweekly. Two weeks after the third vaccination, DTH skin testing is performed for analysis of immune response. At three and six months after first vaccination subjects receive additional "booster" vaccination. Each vaccine contains at least* $25*10^6$ *cells. One third is injected intradermal. Two thirds are administered intravenous. CT=computed tomography, MPM=malignant peritoneal mesothelioma, DTH=delayed type hypersensitivity, WBC=white blood cell, RBC=red blood cell, ANCA=anti-neutrophil cytoplasmic antibody, HIV=human immunodeficiency virus, CRS=cytoreductive surgery, HIPEC=hyperthermic intraperitoneal chemotherapy, DC=dendritic cell*

Fig.2 *DCBI production process. Monocytes are isolated from peripheral blood and are then stimulated to differentiate towards immature dendritic cells. These immature DCs are exposed to PheraLys tumor cell lysate . After further differentiation towards mature DCs, MesoPher vaccinations are given back to the patient. DCs=Dendritic cells*

Fig. 3 *Sample size calculation. Assuming the sensitivity for detecting grade 3 (or higher) toxicity is 99%. Expected prevalence of grade 3 toxicity in the study population is 2.5%.*

For peer review only

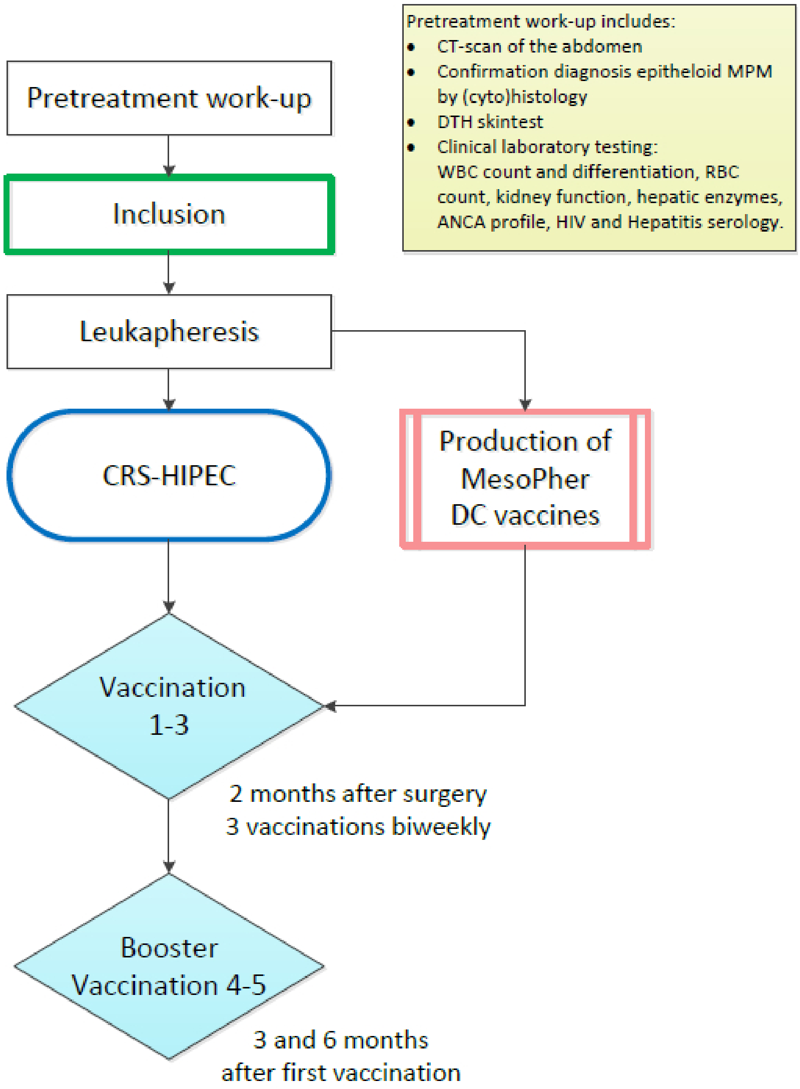

Fig.1 Patient timeline. In total 11 additional visits are required. After informed consent is acquired, screening will take place in the form of full examination and DTH skintest. When patients comply to all criteria, they will undergo leukapheresis for production of dendritic cell vaccine. After two to four weeks patients undergo CRS-HIPEC. Eight to ten weeks after surgery, first vaccination is given, followed by two more vaccinations biweekly. Two weeks after the third vaccination, DTH skin testing is performed for analysis of immune response. At three and six months after first vaccination subjects receive additional "booster" vaccination. Each vaccine contains at least 25*106 cells. One third is injected intradermal. Two thirds are administered intravenous. CT=computed tomography, MPM=malignant peritoneal mesothelioma, DTH=delayed type hypersensitivity, WBC=white blood cell, RBC=red blood cell, ANCA=anti-neutrophil cytoplasmic antibody, HIV=human immunodeficiency virus, CRS=cytoreductive surgery, HIPEC=hyperthermic intraperitoneal chemotherapy, DC=dendritic cell

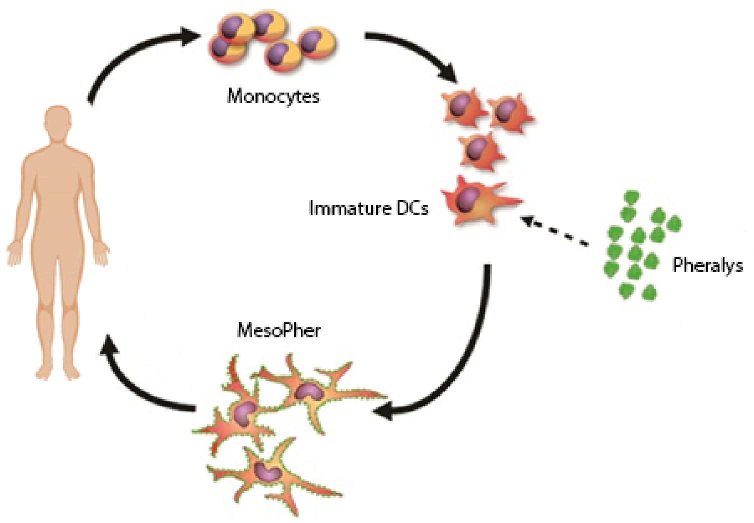

Fig.2 DCBI production process. Monocytes are isolated from peripheral blood and are then stimulated to differentiate towards immature dendritic cells. These immature DCs are exposed to PheraLys tumor cell lysate . After further differentiation towards mature DCs, MesoPher vaccinations are given back to the patient. DCs=Dendritic cells

210x139mm (72 x 72 DPI)

$$N = z_{0.975}^{2} \cdot \frac{sens(1 - sens)}{w^2 \cdot prev}$$

Fig. 3 Sample size calculation. Assuming the sensitivity for detecting grade 3 (or higher) toxicity is 99%. Expected prevalence of grade 3 toxicity in the study population is 2.5%.

BMJ Open

Adjuvant dendritic cell based immunotherapy (DCBI) after cytoreductive surgery (CRS) and hyperthermic intraperitoneal chemotherapy (HIPEC) for peritoneal mesothelioma, a phase II single center open-label clinical trial. Rationale and design of the MESOPEC trial.

Journal:	*BMJ Open*
Manuscript ID	bmjopen-2018-026779.R1
Article Type:	Protocol
Date Submitted by the Author:	06-Feb-2019
Complete List of Authors:	de Boer, Nadine; Erasmus MC, Surgical Oncology<br>van Kooten, Job; Erasmus MC, Surgical Oncology<br>Burger, Jacobus; Erasmus MC, Surgical Oncology<br>Verhoef, Cornelis; Erasmus MC, Surgical Oncology<br>Aerts, Joachim; Erasmus MC, Pulmonary Medicine<br>Madsen, Eva; Erasmus MC, Surgical Oncology
**Primary Subject Heading**:	Oncology
Secondary Subject Heading:	Surgery
Keywords:	Malignant peritoneal mesothelioma, Cytoreductive surgery and hyperthermic intraperitoneal chemotherapy, Dendritic cell based immunotherapy

SCHOLARONE™
Manuscripts

MESOPEC-Trial

Adjuvant dendritic cell based immunotherapy (DCBI) after cytoreductive surgery (CRS) and hyperthermic intraperitoneal chemotherapy (HIPEC) for peritoneal mesothelioma, a phase II single center open-label clinical trial. Rationale and design of the MESOPEC trial.

N.L. de Boer[a], J.P. van Kooten[a], J.W.A. Burger[a], C. Verhoef[a], J.G.J.V. Aerts[b]*, E.V.E. Madsen[a]*

a. Department of Surgical Oncology, Erasmus University Medical Center, Rotterdam, the Netherlands
b. Department of Pulmonary Medicine, Erasmus University Medical Center, Rotterdam, the Netherlands

*contributed equally

Corresponding author:
N.L. de Boer
Erasmus MC
Department of surgical oncology
Dr. Molenwaterplein 40
3015 GD, Rotterdam, the Netherlands
n.deboer@erasmusmc.nl
(+31)010-704 21 25

Word count: 3309

Keywords: Malignant peritoneal mesothelioma, Cytoreductive surgery and hyperthermic intraperitoneal chemotherapy, Dendritic cell based immunotherapy

For peer review only

ABSTRACT

Introduction: Malignant peritoneal mesothelioma (MPM) is an uncommon but aggressive neoplasm and has a strong association with asbestos exposure. MPM has low survival rates of approximately one year even after (palliative) surgery and/or systemic chemotherapy. Recent advances in treatment strategies focusing on cytoreductive surgery (CRS) and hyperthermic intraperitoneal chemotherapy (HIPEC) have resulted in improved median survival of 53 months and a 5-year survival of 47%. However, recurrence rates are high. Current systemic chemotherapy in the adjuvant setting is of limited efficacy, while immunotherapy with dendritic cell based immunotherapy (DCBI) has yielded promising results in murine models with peritoneal mesothelioma and in patients with pleural mesothelioma.

Methods and analysis: The MESOPEC trial is an open-label single center phase II study. The study population are adult patients with histological/cytological confirmed diagnosis of epithelioid malignant peritoneal mesothelioma. Intervention: four to six weeks before CRS-HIPEC a leukapheresis is performed of which the monocytes are used for differentiation to dendritic cells (DCs). Autologous DCs pulsed with allogeneic tumor associated antigens (MesoPher) are re-injected eight to ten weeks after surgery, three times biweekly. Additional booster vaccinations are given at three and six months.

Primary objective is to determine the feasibility of administering DCBI after CRS-HIPEC in patients with malignant peritoneal mesothelioma. Secondary objectives are to assess safety of DCBI in patients with peritoneal mesothelioma and determine whether a specific immunologic response against the tumor occurs as a result of this adjuvant immunotherapy.

Ethics and dissemination: Permission to carry out this study protocol has been granted by the Central Committee on Research Involving Human Subjects (CCMO in Dutch) and the Research Ethics Committee (METC in Dutch). The results of this trial will be submitted for publication in a peer-reviewed journal.

Registration: Netherlands Trial Register: NTR7060. EudraCT: 2017-000897-12.

For peer review only - http://bmjopen.bmj.com/site/about/guidelines.xhtml

Strengths and limitations of this study

- The dendritic cell vaccines used in this protocol can be manufactured on a large scale, because autologous dendritic cells are loaded with allogeneic tumor associated antigens.
- Dendritic cell based immunotherapy has shown to have very limited side effects, especially when compared to systemic chemotherapy.
- This study will provide clinicians and scientists with important information about the immunologic response after dendritic cell vaccination
- Since all patients undergo CRS-HIPEC prior to DCBI therapy, the effect of DCBI treatment must be determined by assessing the immune response and overall clinical condition of each patient.
- In this phase II clinical trial the effect of DCBI on disease free and overall survival cannot be determined, when DCBI is considered safe and feasible, a phase III clinical trial will be conducted to determine the effect on survival.

For peer review only

For peer review only - http://bmjopen.bmj.com/site/about/guidelines.xhtml

1. INTRODUCTION

Malignant peritoneal mesothelioma (MPM) is a highly lethal neoplasm, arising from the serosal lining of the peritoneal cavity. It has a strong association with exposure to asbestos. Non-specific clinical symptoms like weight loss, abdominal pain and distension, contribute to a delay in diagnosis. As a result, the majority of MPM cases are identified at an advanced stage, creating an overall poor life-expectancy of 4-12 months if left untreated.(1) Even after (palliative) surgery and/or systemic chemotherapy, MPM has poor survival of approximately one year.

In recent years treatment focus has shifted towards a more aggressive approach, utilizing cytoreductive surgery (CRS) and hyperthermic intraperitoneal chemotherapy (HIPEC). Patients that underwent this treatment, had a better prognosis with median survival of 53 months and 5-year survival of 47%. (2) However, even after CRS-HIPEC recurrence rates are high with reported median progression-free survival and disease-free survival ranging from 11 to 28 months and 7.2 to 40 months respectively.(1) One explanation is that it is difficult to perform complete cytoreduction, as MPM often grows diffusely throughout the abdominal cavity.(3) But even when macroscopic complete cytoreduction is reached, loco-regional recurrence is often seen. A study that included 108 patients in whom complete or near-complete cytoreduction was achieved, showed local recurrence in 38% of patients after median follow up of 48.8 months.(4)

Effective adjuvant therapies are pressingly needed for above mentioned reasons. Dendritic cell based immunotherapy (DCBI) has shown promising results by harnessing the potency and specificity of the immune system. The first DCBI for mesothelioma was developed in the Erasmus MC Rotterdam and has been tested in murine models with peritoneal mesothelioma and in clinical phase I/II studies for patients with pleural mesothelioma.(5-11) These studies have shown that DCBI induces durable responses and higher survival rates compared to the general mesothelioma population. DC-therapy was well tolerated in these patients without grade 3 or 4 toxicities. Only low grade fever and flu-like symptoms (grade 1-2), were seen for 24 hours after treatment. In a dose escalation phase I trial, the safety of using allogeneic tumor lysate (PheraLys) for the loading of the DCs was assessed. PheraLys is a tumor cell lysate derived from 5 well-characterized cell lines from patients with malignant mesothelioma. Tumor lysate priming strategies may be advantageous in providing the full antigenic repertoire of the tumor and might reduce the possibility of tumor escape by inducing a broader immune response. In this study adverse events were similar to earlier studies and importantly, no severe adverse events were observed. Furthermore, clinical responses were established radiographically and some long time survivors are being observed.

Previous preclinical studies demonstrated that DCBI has the capacity to slow down tumor growth, although tumor load has an important role in survival.(12) Mice had a better outcome when DCs were injected early in tumor development.(5) Mesothelioma cells produce specific cytokines and attract regulatory T-cells that suppress efficient immune responses, indicating that patients with low tumor load have a better functioning

For peer review only - http://bmjopen.bmj.com/site/about/guidelines.xhtml

immune system and better anti-tumor responses.(13) Therefore it is the aim of this trial to treat patients with DCBI after complete macroscopic cytoreduction and HIPEC. The residual disease after cytoreductive surgery is classified using the the 'completeness of cytoreduction' (CCR score). CCR-0 indicates no visible residual tumor and CCR-1 indicates residual tumor nodules ≤ 2.5 mm. CCR-2 indicates residual tumor nodules between 2.5 mm and 2.5 cm. CCR-3 indicates a residual tumor > 2.5 cm. In this study CCR ≤1 is considered as complete macroscopic cytoreduction. However, when complete cytoreduction cannot be achieved during surgery, patients undergo palliative HIPEC followed by DCBI

Main objective of this clinical trial is to determine feasibility of administering adjuvant DCBI after CRS-HIPEC. Secondary objectives are to asses safety and determine if a specific immunologic response against the tumor occurs after DC therapy. When DCBI is considered safe and feasible as adjuvant treatment for patients with MPM, further research (phase III study) is warranted to determine the effect on survival.

2. METHODS AND ANALYSIS

2.1 Study Design

2.1.1 Trial setting

The MESOPEC trial is an open-label, single arm, single center phase II clinical trial. This study is conducted in the Erasmus MC Rotterdam, an academic hospital located in the Netherlands. All patients included in this trial will receive adjuvant DCBI after CRS-HIPEC. Trial registration details are described in Table 1.

2.1.2 Study population

The study population consists of adult patients diagnosed with MPM. Potentially eligible patients will be referred by their local clinician or through self-referral to a medical specialist and member of the study team to discuss the trial and determine eligibility.

In order to participate in the study, patients must meet the following inclusion criteria:

- Histologically or cytological confirmed diagnosis of epithelioid MPM
- WHO-ECOG performance status of 0 or 1
- Normal organ function and adequate bone marrow reserve (absolute neutrophil count >1.0 $*10^9$/l, platelet count >100 $*10^9$/l, Hb >6.0 mmol/l)
- Positive DTH skin test against at least one positive control antigen tetanus toxoid
- Planned start date of vaccination within eight to ten weeks after CRS-HIPEC
- Expected survival prior to surgery must be at least six months
- At least 18 years of age, written informed consent according to ICH-GCP, ability to return to the study center for adequate follow up

For peer review only - http://bmjopen.bmj.com/site/about/guidelines.xhtml

A potential participant who meets any of the following criteria will be excluded from participation in the study:

- Extra abdominal mesothelioma (i.e. metastatic disease)
- Prior cytoreductive surgery
- Prior malignancy other than basal cell carcinoma within ten years of inclusion
- Serious concomitant disease or infection, including HIV or chronic viral hepatitis
- Current use of steroids or other immunosuppressive agents (at least six weeks discontinuation before the first vaccine, with exception of prophylactic usage of dexamethasone during chemotherapy).
- History of auto-immune disease or organ allografts
- Any disease that is considered to constitute an unwarranted high risk for CRS-HIPEC by the surgeon or study coordinator
- Pregnant or lactating women

2.1.3 Patient timeline and additional procedures

Four to six weeks before surgery patients will undergo leukapheresis for dendritic cell vaccine production purposes. At baseline, subjects undergo CRS-HIPEC.

At six weeks after surgery, the investigators will determine if the patient is sufficiently recovered and fit to undergo DCBI. Patients must have adequate bone marrow reserve before DCBI treatment: absolute neutrophil count >1.0 *109/l, platelet count >100*109/l and Hb >6.0mmol/l.

Dendritic cell vaccinations will be given at eight to ten weeks after surgery three times biweekly. Before each vaccination laboratory testing will be performed and results reviewed before injection. Before and after injection vital signs (pulse, blood pressure, blood oxygen saturation and temperature) are determined. Patients are observed in the hospital for two hours after injection. Each vaccine contains at least $25*10^6$ cells. One third of this is injected intradermal, two thirds are administered intravenous. Intradermal injection will be performed in the upper left arm. Intravenous injection will be performed via the vena brachialis in the left arm through a basic peripheral venous catheter. After the third vaccination, delayed type hypersensitivity (DTH) skin test is performed. When DTH skin test result is positive for the dendritic cell vaccine, a 3 mm skin biopsy will be taken for further analyses. At three and six months after the first vaccination, two additional booster vaccinations will be given. Additional to study-related treatment, patients receive standard care and follow up required after CRS-HIPEC.

Total duration of the treatment protocol is eight to nine months. In total, 11 additional visits are required to adhere to the study protocol. For the production of DCBI patients have to undergo leukapheresis. For the purpose of immune-monitoring, additional blood samples will be collected at seven moments during the study. (fig.1)

2.1.4 Dendritic cell vaccine production

Dendritic cells are derived from peripheral blood mononuclear cells (PBMCs), by differentiating monocytes towards immature dendritic cells using specific cytokines. Immature dendritic cells are known for their high antigen uptake potential. Therefore they are exposed to tumor specific antigens (TAA) in a co-culture with

For peer review only - http://bmjopen.bmj.com/site/about/guidelines.xhtml

allogeneic mesothelioma cell lysate. This lysate (PheraLys) is derived from five well specified mesothelioma cell lines. After exposure to PheraLys the immature dendritic cells are differentiated towards mature dendritic cells, which are ready to activate the immune system in vivo (MesoPher). (fig.2)

2.1.5 Withdrawal of individual subjects

Subjects can leave the study at any time for any reason if they wish to do so without any consequences. The investigator can decide to withdraw a subject from the study for urgent medical reasons. Should a participant withdraw from the trial, then every effort will be made to obtain follow-up data, with the permission of the patient.

The investigators also have the right to withdraw patients from the study if one or more of the following events occur:

- Significant protocol violation or noncompliance on the part of the patient or investigator.
- Refusal of the patient to continue treatment or observations.
- Any change in the condition of the patient that justifies discontinuation of treatment.
- Decision by the study coordinator that MesoPher does not comply to quality requirements (advice of Qualified Person)
- Decision by the study coordinator that termination is in the patient's best medical interest.
- Unrelated medical illness or complication.
- Serious logistic problems of practical problems in cleanroom

2.2 Objectives and Analysis

Primary objective

Primary objective is to determine feasibility of using DCBI after CRS-HIPEC in patients with peritoneal mesothelioma. DCBI after CRS-HIPEC is deemed feasible when at least 75% of patients that are included in this trial complete the full treatment schedule. This cut-off is based on the fact that currently around 75% of patients undergoing CRS-HIPEC are fit to undergo adjuvant therapy, such as systemic chemotherapy.

Secondary objectives

Secondary objectives are to asses safety of DCBI therapy after CRS-HIPEC and determine whether a specific immunologic response occurs due to dendritic cell vaccination

Safety

Previous clinical studies have shown that injection with tumor lysate-pulsed autologous DCs was overall well tolerated without systemic toxicity, with the exception of low-grade flu-like symptoms like fever and rigors. No grade 3 or 4 toxicity was observed.(11) However, safety and tolerability after major surgery has yet to be determined.

For peer review only - http://bmjopen.bmj.com/site/about/guidelines.xhtml

The administration of autologous cells that have been loaded with allogeneic human materials is a potential health risk. Because not the lysate itself is administered to the patients, but only after it has been processed by autologous dendritic cells, risks are expected to be limited.
The necessary sample size for the detection of grade 3 toxicity is calculated to be 20 patients (fig.3). All adverse events (AEs), serious adverse events (SAEs) and suspected unexpected serious adverse reactions (SUSARS) will be monitored and reported by the sponsor.

Immune response
Assessment of immune responses will be conducted on three levels in all treated patients; 1. responses that mark successful vaccination, 2. enhanced frequencies of tumor-specific T cells in peripheral blood samples, and 3. frequency shifts in other immune cell subsets.

1. Keyhole Limpet Hemocyanin (KLH) is added to the vaccines as a surrogate marker. With the use of KLH, we will assess whether an immune response against the vaccine has occurred and whether this response persists. KLH is known to induce a specific adaptive immune response readily detectable in sera (antibody response) and skin tests (cellular response) of vaccinated individuals. Serum samples will be collected before, during and after DCBI as well as at selected intervals during follow-up. Humoral responses to KLH will be detected using enzyme-linked immune sorbent assay (ELISA). Furthermore, patients will undergo a delayed type hypersensitivity (DTH) skin test before and after DCBI. DTH responses will be evaluated for local inflammation after 48 hours and 3 mm punch biopsies will be collected in case inflammation occurs. These biopsies will subsequently be used for in situ immunostainings of i.e. DC, myeloid derived suppressor cells (MDSC), and CD8+ T cells.

2. To assess vaccine-induced frequencies of tumor-specific T cells, we will conduct a potency assay in accordance with the "Guideline on potency testing of cell based immunotherapy medicinal products for the treatment of cancer" as provided by the European Medicine Agency (EMA) in 2016 (EMA/CHMP/BWP/271475/2006 rev.1). The proposed assay can shortly be described as a co-culture of T cells isolated from pre- and post-treatment peripheral blood samples with autologous DCs loaded with autologous tumor lysate. Subsequently, we will measure T cell proliferation and activation markers via multicolor flow cytometry.

3. Phenotypical analysis of immune cell subsets will be conducted using flow cytometry to detect vaccine-induced changes in the frequencies of >100 immune cell(subsets) that represent distinct lineages and/or express different levels of activation, differentiation and co-signaling markers . Staining of fresh patient material at different time points will allow enumeration of different immune cells throughout therapy. Subsequent bulk analysis of frozen material focusses solely on an extensive array of T cell, MDSC and DC markers. Combination of these readouts allows for the generation of immune profiles for individual patients. The analysis of these profiles in turn will allow for the

For peer review only - http://bmjopen.bmj.com/site/about/guidelines.xhtml

determination of prospective markers and better stratification of patient populations suited for vaccination therapy.

Statistical analysis

The statistical analyses/data summaries will be performed using SPSS. Other tools may be used for exploratory summaries and graphical presentations.

Primary endpoint is to determine the feasibility of administering DCBI after CRS-HIPEC in patients with malignant peritoneal mesothelioma. Based on previous results it is known that from all patients with colorectal carcinoma that underwent CRS-HIPEC about 25% is not able to receive adjuvant systemic therapy due to postoperative complications. Feasibility is set if 15 of 20 patients (75%) were able to undergo leukapheresis successfully, production of PheraLys and dendritic cell vaccines was successful and when patients were able to complete the full adjuvant treatment schedule.

2.3 Patient and public involvement

The Dutch patient association for patients with mesothelioma "Instituut Asbestslachtoffers" and the Erasmus MC Rotterdam have worked together closely for years on the development of dendritic cell immunotherapy for mesothelioma. The patient association has received a copy of the study protocol to comment on the research question and outcome measures and also received the patient information folder to comment on patients preference and clarity of the information folder. During the study, feedback is provided on the inclusion rate and patient experiences. The results of the study will also be communicated to the patient association, which can then distribute them among their members.

3 ETHICS AND DISSEMINATION

Permission to implement this study protocol has been granted by the Central Committee on Research Involving Human Subjects (CCMO in Dutch) and the Research Ethics Committee (METC in Dutch) of the Erasmus MC. The study will be conducted in compliance with the 'Medical Research Involving Human Subjects Act' (WMO) and according to the principles of the Declaration of Helsinki (64th World Medical Association General Assembly, Fortaleza, Brazil, October 2013).

If protocol modifications occur, the new protocol has to be approved by the Central Committee on Research Involving Human Subjects and the Research Ethics Committee of the Erasmus MC before they can be implemented. Data collection, data assessment and data analysis will be performed according to the local guidelines for data management of the Erasmus MC.

To generate more awareness of this current study and to increase referrals of potential study candidates to the Erasmus MC, a short Dutch summary of the study will be submitted to The Dutch Journal for Oncology (NTVO in Dutch). Also, presentations about the study have been given at the Dutch Society of Surgery meeting and the Peritoneal Surface Oncology Group International meeting in Paris.

The results of this clinical trial will be submitted for publication in a peer-reviewed scientific journal.

For peer review only - http://bmjopen.bmj.com/site/about/guidelines.xhtml

The investigator will permit auditors to carry out site visits to audit the compliance with regulatory guidelines. Similar auditing procedures may be conducted by agents of any regulatory body reviewing the results of this study.

The sponsor will submit yearly safety report to the accredited METC, competent authority, and competent authorities of the concerned Member States. This safety report consists of: (1) A list of all suspected (unexpected or expected) serious adverse reactions. (2) a report concerning the safety of the subjects, consisting of a complete safety analysis and an evaluation of the balance between the efficacy and the harmfulness of the medicine under investigation.

Within one year after the end of the study, the investigator/sponsor will submit a final study report with the results of the study, including any publications/abstracts of the study, to the accredited METC and the Competent Authority.

Currently three patients are included in the study protocol. Final patient is expected to be included at the end of 2020. First results are expected in 2021.

4 DISCUSSION

The main objective of the MESOPEC trial is to determine feasibility of DCBI as adjuvant treatment after CRS-HIPEC in patients with MPM. Secondary objectives are to assess safety and to monitor the immune response after DCBI. The MESOPEC trial is the first clinical trial offering adjuvant dendritic cell immunotherapy to patients with MPM. So far (neo)adjuvant systemic chemotherapy has shown no benefit on surgical or oncological outcome for MPM.(14) Systemic chemotherapy, using cisplatin and pemetrexed, is standard treatment in pleural mesothelioma and has been applied to patients with peritoneal mesothelioma. This has shown limited efficacy, considerable toxicity and even mortality, making it unfit for the treatment of MPM. (15)

The DCBI used in this current trial consists of personalized dendritic cell vaccines, produced with autologous dendritic cells that are loaded with tumor associated antigens derived from allogeneic tumor cell lysate. This treatment approach has multiple advantages. The biggest advantage of this strategy is that it is possible to produce personalized anti-cancer vaccines on a scale sufficient for clinical implementation in larger groups of patients. Another advantage is that so far DCBI has shown no severe side effects and therefore causes little morbidity especially when compared to current other adjuvant treatment options, like systemic chemotherapy.

Analyses of tissue and blood samples that are collected throughout the study will provide valuable information for scientists and clinicians regarding the immunologic response after DCBI. Furthermore, potential of DCBI can be tested in vitro by culturing tumor cell lines derived from tumor tissue obtained during CRS. By doing so, in the future patients that will respond to immunotherapy can be identified before starting treatment.

Currently, CRS-HIPEC is the gold standard for a selected group of patients suffering from MPM. Eligibility for CRS-HIPEC is dependent on several clinical aspects such as ‘peritoneal carcinoma index’ (PCI), histological

For peer review only - http://bmjopen.bmj.com/site/about/guidelines.xhtml

subtype and overall patient health condition. Previous studies have shown that outcome after CRS-HIPEC strongly depends on the completeness of cytoreduction.(1) Unfortunately it is very difficult to achieve complete cytoreduction in patients with high PCI-score. It has been reported by others that immunotherapy is possibly more effective in patients with low tumor load. Therefore, in this study DCBI is given as adjuvant treatment after CRS-HIPEC. However, if a significant clinical effect can be achieved, in the future DCBI might be used as a neoadjuvant therapy making CRS-HIPEC with complete cytoreduction available for a larger number of patients.

We acknowledge the fact that this study has limitations. One limitation of this study is the small number of patients that will be included. Given the rarity of MPM, it is difficult to include large numbers of patients. However the sample size of this current study, should be sufficient to determine the feasibility and safety of adjuvant DCBI after CRS-HIPEC.

Another limitation of this study design is that it is not possible to determine radiological response to DCBI treatment. After debulking surgery, MPM will not be detectable on CAT-scans. Therefore the response to DCBI treatment must be determined by assessing the immune response and overall clinical condition of each patient. Clinical effect of DCBI on overall survival can only be determined after a longer period of follow up.

If DCBI is considered feasible as adjuvant treatment for MPM, a larger phase III clinical trial should be conducted to determine the effect on surgical and oncological outcome. Because MPM incidence in the Netherlands alone is very low, this clinical trial would have to be conducted internationally.

5 ACKNOWLEDGEMENTS

We would like to thank the Dutch patient association for patients with mesothelioma for their detailed advice and guidance during the design of this study.

6 AUTHORS CONTRIBUTIONS

NB and JK drafted this manuscript. NB, JB, JA, CV and EM drafted the original study protocol and revised the manuscript. CV, JA and EM initiated the trial and supervised the drafting of the study protocol and manuscript. EM acquired funding for implementation of the trial protocol and is the primary clinical investigator. All authors approved the final manuscript.

7 COMPETING INTERESTS

None declared.

8 FUNDING STATEMENT

This work was supported by: The Dutch Cancer Society (in Dutch: KWF Kankerbestrijding), grant number: 10246,

For peer review only - http://bmjopen.bmj.com/site/about/guidelines.xhtml

Stichting Coolsingel, grant number: 482

9 REFERENCES

1. Helm JH, Miura JT, Glenn JA, et al. Cytoreductive surgery and hyperthermic intraperitoneal chemotherapy for malignant peritoneal mesothelioma: a systematic review and meta-analysis. Ann Surg Oncol. 2015;22(5):1686-93.

2. Yan TD, Deraco M, Baratti D, et al. Cytoreductive surgery and hyperthermic intraperitoneal chemotherapy for malignant peritoneal mesothelioma: multi-institutional experience. J Clin Oncol. 2009;27(36):6237-42.

3. Yan TD, Welch L, Black D, et al. A systematic review on the efficacy of cytoreductive surgery combined with perioperative intraperitoneal chemotherapy for diffuse malignancy peritoneal mesothelioma. Ann Oncol. 2007;18(5):827-34.

4. Baratti D, Kusamura S, Cabras AD, et al. Diffuse malignant peritoneal mesothelioma: long-term survival with complete cytoreductive surgery followed by hyperthermic intraperitoneal chemotherapy (HIPEC). Eur J Cancer. 2013;49(15):3140-8.

5. Hegmans JP, Hemmes A, Aerts JG, et al. Immunotherapy of murine malignant mesothelioma using tumor lysate-pulsed dendritic cells. Am J Respir Crit Care Med. 2005;171(10):1168-77.

6. Hegmans JP, Veltman JD, Lambers ME, et al. Consolidative dendritic cell-based immunotherapy elicits cytotoxicity against malignant mesothelioma. Am J Respir Crit Care Med. 2010;181(12):1383-90.

7. Veltman JD, Lambers ME, van Nimwegen M, et al. Low-dose cyclophosphamide synergizes with dendritic cell-based immunotherapy in antitumor activity. J Biomed Biotechnol. 2010;2010:798467.

8. Cornelissen R, Heuvers ME, Maat AP, et al. New roads open up for implementing immunotherapy in mesothelioma. Clin Dev Immunol. 2012;2012:927240.

9. Heuvers ME, Wisnivesky J, Stricker BH, et al. Generalizability of results from the National Lung Screening Trial. Eur J Epidemiol. 2012;27(9):669-72.

10. Cornelissen R, Lievense LA, Heuvers ME, et al. Dendritic cell-based immunotherapy in mesothelioma. Immunotherapy. 2012;4(10):1011-22.

11. Cornelissen R, Hegmans JP, Maat AP, et al. Extended Tumor Control after Dendritic Cell Vaccination with Low-Dose Cyclophosphamide as Adjuvant Treatment in Patients with Malignant Pleural Mesothelioma. Am J Respir Crit Care Med. 2016;193(9):1023-31.

12. Anguille S, Smits EL, Lion E, et al. Clinical use of dendritic cells for cancer therapy. Lancet Oncol. 2014;15(7):e257-67.

13. Hegmans JP, Hemmes A, Hammad H,et al. Mesothelioma environment comprises cytokines and T-regulatory cells that suppress immune responses. Eur Respir J. 2006;27(6):1086-95.

14. Deraco M, Baratti D, Hutanu I, et al. The role of perioperative systemic chemotherapy in diffuse malignant peritoneal mesothelioma patients treated with cytoreductive surgery and hyperthermic intraperitoneal chemotherapy. Ann Surg Oncol. 2013;20(4):1093-100.

For peer review only - http://bmjopen.bmj.com/site/about/guidelines.xhtml

15. Janne PA, Wozniak AJ, Belani CP, et al. Open-label study of pemetrexed alone or in combination with cisplatin for the treatment of patients with peritoneal mesothelioma: outcomes of an expanded access program. Clin Lung Cancer. 2005;7(1):40-6.

Fig.1 *Patient timeline. In total 11 additional visits are required. After informed consent is acquired, screening will take place in the form of full examination and DTH skintest. When patients comply to all criteria, they will undergo leukapheresis for production of dendritic cell vaccine. After two to four weeks patients undergo CRS-HIPEC. Eight to ten weeks after surgery, first vaccination is given, followed by two more vaccinations biweekly. Two weeks after the third vaccination, DTH skin testing is performed for analysis of immune response. At three and six months after first vaccination subjects receive additional "booster" vaccination. Each vaccine contains at least* $25*10^6$ *cells. One third is injected intradermal. Two thirds are administered intravenous. CT=computed tomography, MPM=malignant peritoneal mesothelioma, DTH=delayed type hypersensitivity, WBC=white blood cell, RBC=red blood cell, ANCA=anti-neutrophil cytoplasmic antibody, HIV=human immunodeficiency virus, CRS=cytoreductive surgery, HIPEC=hyperthermic intraperitoneal chemotherapy, DC=dendritic cell*

Fig.2 *DCBI production process. Monocytes are isolated from peripheral blood and are then stimulated to differentiate towards immature dendritic cells. These immature dendritic cells are exposed to PheraLys tumor cell lysate . After further differentiation towards mature dendritic cells, MesoPher vaccinations are given back to the patient.*

Fig. 3 *Sample size calculation. Assuming the sensitivity for detecting grade 3 (or higher) toxicity is 99%. Expected prevalence of grade 3 toxicity in the study population is 2.5%.*

For peer review only

For peer review only - http://bmjopen.bmj.com/site/about/guidelines.xhtml

Table 1. World Health Organization Trial Registration Data Set

Primary registry and trial identifying number	EudraCT number: 2017-000897-12<br>Netherlands Trial Register: NTR7060
Date of registration in primary registry	October 2017
Protocol version	Protocol version 3.0, date 31-10-2017
SPIRIT guidelines data set for clinical trials	See Supplementary file
Source(s) of monetary or material support	Erasmus University Medical Center, Rotterdam, the Netherlands<br>Dutch Cancer Society (KWF Kankerbestrijding)<br>Stichting Coolsingel
Primary sponsor	Erasmus University Medical Center, Rotterdam, the Netherlands
Secondary sponsors	Dutch cancer society (KWF Kankerbestrijding)<br>Stichting Coolsingel
Contact for public queries	N.L. de Boer, study coordinator<br>Department of surgical oncology<br>Erasmus MC, Rotterdam, the Netherlands<br>n.deboer@erasmusmc.nl , (+31)010-704 21 25<br><br>J.P. van Kooten, study coordinator<br>Department of surgical oncology<br>Erasmus MC, Rotterdam, the Netherlands<br>j.kooten@erasmusmc.nl, (+31)010-704 21 25
Contact for scientific queries	E.V.E. Madsen, principal investigator<br>Department of surgical oncology<br>Erasmus MC, Rotterdam, the Netherlands<br>e.madsen@erasmusmc.nl, (+31)010-704 10 82
Public title	Adjuvant dendritic cell based immunotherapy (DCBI) after cytoreductive surgery (CRS) and hyperthermic intraperitoneal chemotherapy (HIPEC) for peritoneal mesothelioma: the MESOPEC-trial.
Scientific title	Adjuvant dendritic cell based immunotherapy (DCBI) after cytoreductive surgery (CRS) and hyperthermic intraperitoneal chemotherapy (HIPEC) for peritoneal mesothelioma. A phase II single center open-label clinical trial.

For peer review only

Countries of recruitment	The Netherlands
Health conditions or problems studied	Malignant peritoneal mesothelioma
Interventions	Vaccination with autologous dendritic cells loaded with allogeneic mesothelioma specific tumor antigens, after standard care (CRS-HIPEC)
Key inclusion and exclusion criteria	Inclusion Criteria:<br>Confirmed diagnosis of epithelial peritoneal mesothelioma.<br>WHO-ECOG performance status 0-1, expected survival at least six months.<br>Adequate organ function and bone marrow reserves.<br>Positive delayed type hypersensitivity skin test for positive control antigen.<br><br>Exclusion criteria:<br>Extra-abdominal disease/metastatic disease.<br>Current use of steroids or other immunosuppressive agents.<br>Prior cytoreductive surgery.<br>Prior malignancy other than basal cell carcinoma within ten years of inclusion.<br>Patients with a known allergy to shellfish<br>Serious chronic or acute illness considered to constitute unwarranted high risk for CRS-HIPEC or dendritic cell treatment.<br>Pregnant or lactating women.
Study type	Open label single center phase II study
Date of first enrolment	March 2018
Target sample size	20
Recruitment status	Recruiting
Primary outcome	Feasibility (DCBI therapy is considered feasible when 75% of patients enrolled in this study are able to receive and finish dendritic cell vaccination after CRS-HIPEC)
Key secondary outcome(s)	Safety<br>Immunologic response after dendritic cell vaccination

For peer review only - http://bmjopen.bmj.com/site/about/guidelines.xhtml

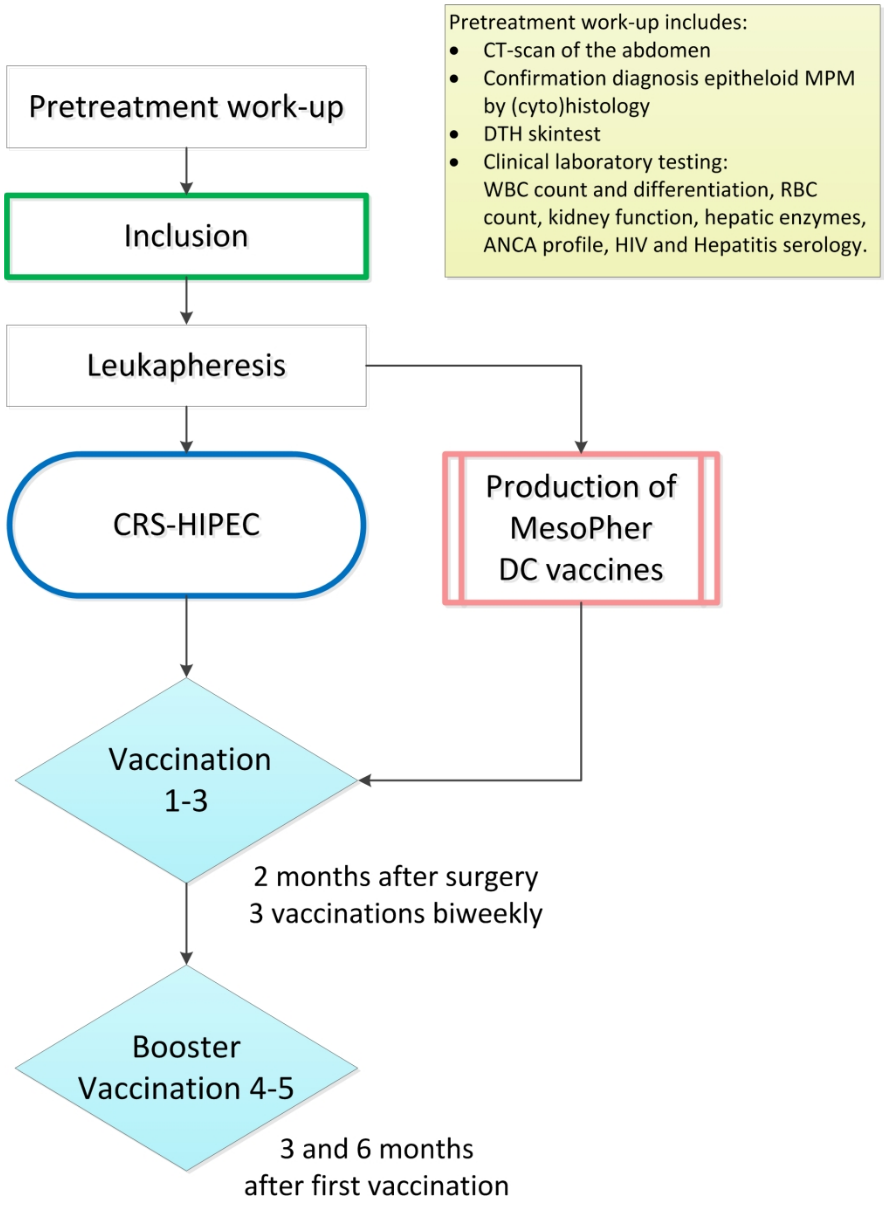

Fig.1 Patient timeline. In total 11 additional visits are required. After informed consent is acquired, screening will take place in the form of full examination and DTH skintest. When patients comply to all criteria, they will undergo leukapheresis for production of dendritic cell vaccine. After two to four weeks patients undergo CRS-HIPEC. Eight to ten weeks after surgery, first vaccination is given, followed by two more vaccinations biweekly. Two weeks after the third vaccination, DTH skin testing is performed for analysis of immune response. At three and six months after first vaccination subjects receive additional “booster” vaccination. Each vaccine contains at least 25*106 cells. One third is injected intradermal. Two thirds are administered intravenous. CT=computed tomography, MPM=malignant peritoneal mesothelioma, DTH=delayed type hypersensitivity, WBC=white blood cell, RBC=red blood cell, ANCA=anti-neutrophil cytoplasmic antibody, HIV=human immunodeficiency virus, CRS=cytoreductive surgery, HIPEC=hyperthermic intraperitoneal chemotherapy, DC=dendritic cell

161x220mm (300 x 300 DPI)

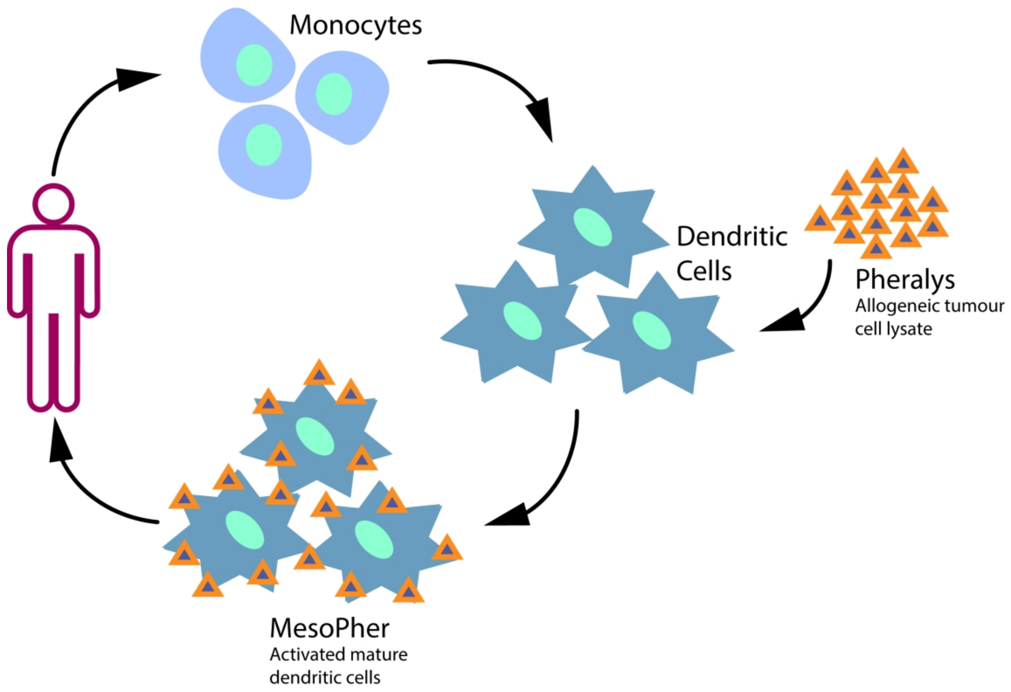

Fig.2 DCBI production process. Monocytes are isolated from peripheral blood and are then stimulated to differentiate towards immature dendritic cells. These immature dendritic cells are exposed to PheraLys tumor cell lysate . After further differentiation towards mature dendritic cells, MesoPher vaccinations are given back to the patient.

$$N = z_{0.975}^{2} \cdot \frac{sens(1 - sens)}{w^{2} \cdot prev}$$

For peer review only

SPIRIT

STANDARD PROTOCOL ITEMS: RECOMMENDATIONS FOR INTERVENTIONAL TRIALS

SPIRIT 2013 Checklist: Recommended items to address in a clinical trial protocol and related documents*

Section/item	Item No	Description
**Administrative information**		
Title	1	Descriptive title identifying the study design, population, interventions, and, if applicable, trial acronym<br>This can be found on the title page of the manuscript, page 1.
Trial registration	2a	Trial identifier and registry name. If not yet registered, name of intended registry<br>This is stated in the World Health Organization Trial Registration Data Set, Table 1.
	2b	All items from the World Health Organization Trial Registration Data Set<br>This is stated in the World Health Organization Trial Registration Data Set, Table 1.
Protocol version	3	Date and version identifier<br>This is stated in the World Health Organization Trial Registration Data Set, Table 1.
Funding	4	Sources and types of financial, material, and other support<br>This is stated in the World Health Organization Trial Registration Data Set, Table 1.
Roles and responsibilities	5a	Names, affiliations, and roles of protocol contributors<br>This can be found on the title page of the manuscript, page 1, and on page 11 in Authors contributions.
	5b	Name and contact information for the trial sponsor<br>This is stated in the World Health Organization Trial Registration Data Set, Table 1.
	5c	Role of study sponsor and funders, if any, in study design; collection, management, analysis, and interpretation of data; writing of the report; and the decision to submit the report for publication, including whether they will have ultimate authority over any of these activities.<br>Not applicable, study sponsors and funders did not have a role in study design, and will not have a role in data management or analysis.

For peer review only

	5d	Composition, roles, and responsibilities of the coordinating centre, steering committee, endpoint adjudication committee, data management team, and other individuals or groups overseeing the trial, if applicable (see Item 21a for data monitoring committee)<br>Not applicable.

Introduction

Background and rationale	6a	Description of research question and justification for undertaking the trial, including summary of relevant studies (published and unpublished) examining benefits and harms for each intervention<br>This is stated in the Introduction of the manuscript, page 4.
	6b	Explanation for choice of comparators<br>Not applicable
Objectives	7	Specific objectives or hypotheses<br>This is stated in section 2.2 Objectives and Analysis of the manuscript, page 7.
Trial design	8	Description of trial design including type of trial (eg, parallel group, crossover, factorial, single group), allocation ratio, and framework (eg, superiority, equivalence, noninferiority, exploratory)<br>This is stated in section 2.1 Study design of the manuscript, page 5.

Methods: Participants, interventions, and outcomes

Study setting	9	Description of study settings (eg, community clinic, academic hospital) and list of countries where data will be collected. Reference to where list of study sites can be obtained<br>The MESOPEC trial is an open-label, single arm, single center phase II clinical trial. This study is conducted in the Erasmus MC Rotterdam, an academic hospital located in the Netherlands. As is also mentioned under 2.1.1 Study design, page 5.
Eligibility criteria	10	Inclusion and exclusion criteria for participants. If applicable, eligibility criteria for study centres and individuals who will perform the interventions (eg, surgeons, psychotherapists)<br>Inclusion and Exclusion criteria are stated in section 2.1.2 Study population of the manuscript, page 5-6
Interventions	11a	Interventions for each group with sufficient detail to allow replication, including how and when they will be administered<br>This is stated in section 2.1 Study design of the manuscript, page 5-6.
	11b	Criteria for discontinuing or modifying allocated interventions for a given trial participant (eg, drug dose change in response to harms, participant request, or improving/worsening disease)<br>This is stated in section 2.1.5 Withdrawal of individual subjects, page 7

For peer review only

	11c	Strategies to improve adherence to intervention protocols, and any procedures for monitoring adherence (eg, drug tablet return, laboratory tests)<br>Not applicable
	11d	Relevant concomitant care and interventions that are permitted or prohibited during the trial<br>Current use of steroids or other immunosuppressive agents is prohibited, as is stated in the Inclusion and Exclusion criteria on page 5-6
Outcomes	12	Primary, secondary, and other outcomes, including the specific measurement variable (eg, systolic blood pressure), analysis metric (eg, change from baseline, final value, time to event), method of aggregation (eg, median, proportion), and time point for each outcome. Explanation of the clinical relevance of chosen efficacy and harm outcomes is strongly recommended<br>This is stated in section 2.2 Objectives and Analysis of the manuscript, page 7-9.
Participant timeline	13	Time schedule of enrolment, interventions (including any run-ins and washouts), assessments, and visits for participants. A schematic diagram is highly recommended (see Figure)<br>This is described in Figure 1 'Patients timeline'.
	14	Estimated number of participants needed to achieve study objectives and how it was determined, including clinical and statistical assumptions supporting any sample size calculations<br>This is described in Figure 3 'Sample size calculation'.
Recruitment	15	Strategies for achieving adequate participant enrolment to reach target sample size<br>This is described in section 3 Ethics and Dissemination of the manuscript, page 9.

Methods: Assignment of interventions (for controlled trials)

Allocation:

Sequence generation	16a	Method of generating the allocation sequence (eg, computer-generated random numbers), and list of any factors for stratification. To reduce predictability of a random sequence, details of any planned restriction (eg, blocking) should be provided in a separate document that is unavailable to those who enrol participants or assign interventions<br>Not applicable
Allocation concealment mechanism	16b	Mechanism of implementing the allocation sequence (eg, central telephone; sequentially numbered, opaque, sealed envelopes), describing any steps to conceal the sequence until interventions are assigned<br>Not applicable

For peer review only - http://bmjopen.bmj.com/site/about/guidelines.xhtml

Implementation	16c	Who will generate the allocation sequence, who will enrol participants, and who will assign participants to interventions<br>Not applicable
Blinding (masking)	17a	Who will be blinded after assignment to interventions (eg, trial participants, care providers, outcome assessors, data analysts), and how<br>Not applicable
	17b	If blinded, circumstances under which unblinding is permissible, and procedure for revealing a participant's allocated intervention during the trial<br>Not applicable
**Methods: Data collection, management, and analysis**		
Data collection methods	18a	Plans for assessment and collection of outcome, baseline, and other trial data, including any related processes to promote data quality (eg, duplicate measurements, training of assessors) and a description of study instruments (eg, questionnaires, laboratory tests) along with their reliability and validity, if known. Reference to where data collection forms can be found, if not in the protocol<br>This is described in section 3 Ethics and Dissemination of the manuscript, page 9-10.
	18b	Plans to promote participant retention and complete follow-up, including list of any outcome data to be collected for participants who discontinue or deviate from intervention protocols<br>This is stated in section 2.1.5 Withdrawal of individual subjects, page 7.
Data management	19	Plans for data entry, coding, security, and storage, including any related processes to promote data quality (eg, double data entry; range checks for data values). Reference to where details of data management procedures can be found, if not in the protocol<br>This is described in section 3 Ethics and Dissemination of the manuscript, page 9-10.
Statistical methods	20a	Statistical methods for analysing primary and secondary outcomes. Reference to where other details of the statistical analysis plan can be found, if not in the protocol<br>This is stated in section 2.2 Objectives and Analysis of the manuscript, page 7-9.
	20b	Methods for any additional analyses (eg, subgroup and adjusted analyses)<br>This is stated in section 2.2 Objectives and Analysis of the manuscript, page 7-9.

	20c	Definition of analysis population relating to protocol non-adherence (eg, as randomised analysis), and any statistical methods to handle missing data (eg, multiple imputation)<br>This is stated in section 2.1.5 Withdrawal of individual subjects, page 7.
**Methods: Monitoring**		
Data monitoring	21a	Composition of data monitoring committee (DMC); summary of its role and reporting structure; statement of whether it is independent from the sponsor and competing interests; and reference to where further details about its charter can be found, if not in the protocol. Alternatively, an explanation of why a DMC is not needed<br>No DSMB was installed for this study. The the Central Committee on Research Involving Human Subjects (CCMO in Dutch) and the Research Ethics Committee (METC in Dutch) of the Erasmus MC agreed with this decision.
	21b	Description of any interim analyses and stopping guidelines, including who will have access to these interim results and make the final decision to terminate the trial<br>Not applicable
Harms	22	Plans for collecting, assessing, reporting, and managing solicited and spontaneously reported adverse events and other unintended effects of trial interventions or trial conduct<br>This is stated in section 2.2 Objectives and Analysis under 'safety' on page 7-9, and in section '3 Ethics and Dissemination' on page 9-10.
Auditing	23	Frequency and procedures for auditing trial conduct, if any, and whether the process will be independent from investigators and the sponsor<br>Not applicable
**Ethics and dissemination**		
Research ethics approval	24	Plans for seeking research ethics committee/institutional review board (REC/IRB) approval<br>This is described in section 3 Ethics and Dissemination of the manuscript, page 9-10.
Protocol amendments	25	Plans for communicating important protocol modifications (eg, changes to eligibility criteria, outcomes, analyses) to relevant parties (eg, investigators, REC/IRBs, trial participants, trial registries, journals, regulators)<br>This is described in section 3 Ethics and Dissemination of the manuscript, page 9-10.
Consent or assent	26a	Who will obtain informed consent or assent from potential trial participants or authorised surrogates, and how (see Item 32)<br>This is described in Figure 1 Patients timeline.

For peer review only - http://bmjopen.bmj.com/site/about/guidelines.xhtml

	26b	Additional consent provisions for collection and use of participant data and biological specimens in ancillary studies, if applicable<br>This is described in the patient information folder, that every patient will receive and which is approved by the Central Committee on Research Involving Human Subjects (CCMO in Dutch) and the Research Ethics Committee (METC in Dutch) of the Erasmus MC. Since this is a single center study, performed in the Netherlands, the approved patient information folder is in Dutch. Therefore it is not added to the manuscript.
Confidentiality	27	How personal information about potential and enrolled participants will be collected, shared, and maintained in order to protect confidentiality before, during, and after the trial<br>This is described in the patient information folder, that every patient will receive and which is approved by the Central Committee on Research Involving Human Subjects (CCMO in Dutch) and the Research Ethics Committee (METC in Dutch) of the Erasmus MC. Since this is a single center study, performed in the Netherlands, the approved patient information folder is in Dutch. Therefore it is not added to the manuscript.
Declaration of interests	28	Financial and other competing interests for principal investigators for the overall trial and each study site<br>Not applicable
Access to data	29	Statement of who will have access to the final trial dataset, and disclosure of contractual agreements that limit such access for investigators<br>This is described in the patient information folder, that every patient will receive and which is approved by the Central Committee on Research Involving Human Subjects (CCMO in Dutch) and the Research Ethics Committee (METC in Dutch) of the Erasmus MC. Since this is a single center study, performed in the Netherlands, the approved patient information folder is in Dutch. Therefore it is not added to the manuscript.
Ancillary and post-trial care	30	Provisions, if any, for ancillary and post-trial care, and for compensation to those who suffer harm from trial participation<br>This is described in the patient information folder, that every patient will receive and which is approved by the Central Committee on Research Involving Human Subjects (CCMO in Dutch) and the Research Ethics Committee (METC in Dutch) of the Erasmus MC. Since this is a single center study, performed in the Netherlands, the approved patient information folder is in Dutch. Therefore it is not added to the manuscript.
Dissemination policy	31a	Plans for investigators and sponsor to communicate trial results to participants, healthcare professionals, the public, and other relevant groups (eg, via publication, reporting in results databases, or other data sharing arrangements), including any publication restrictions<br>This is described in section 3 Ethics and Dissemination of the manuscript, page 9-10.

For peer review

For peer review only - http://bmjopen.bmj.com/site/about/guidelines.xhtml

	31b	Authorship eligibility guidelines and any intended use of professional writers<br>Not applicable
	31c	Plans, if any, for granting public access to the full protocol, participant-level dataset, and statistical code<br>Not applicable

Appendices

Informed consent materials	32	Model consent form and other related documentation given to participants and authorised surrogates<br>The consent forms are approved by the Central Committee on Research Involving Human Subjects (CCMO in Dutch) and the Research Ethics Committee (METC in Dutch) of the Erasmus MC. Since this is a single center study performed in the Netherlands, the approved consent forms are in Dutch. Therefore these are not added to the manuscript.
Biological specimens	33	Plans for collection, laboratory evaluation, and storage of biological specimens for genetic or molecular analysis in the current trial and for future use in ancillary studies, if applicable<br>This is stated in section 2.2 Objectives and Analysis of the manuscript, page 7-9

*It is strongly recommended that this checklist be read in conjunction with the SPIRIT 2013 Explanation & Elaboration for important clarification on the items. Amendments to the protocol should be tracked and dated. The SPIRIT checklist is copyrighted by the SPIRIT Group under the Creative Commons "Attribution-NonCommercial-NoDerivs 3.0 Unported" license.

For peer review only
